# Impact of Exposure to Fenitrothion on Vital Organs in Rats

**DOI:** 10.1155/2016/5609734

**Published:** 2016-11-16

**Authors:** Rasha Abdel-Ghany, Ebaa Mohammed, Shimaa Anis, Waleed Barakat

**Affiliations:** ^1^Department of Pharmacology & Toxicology, Faculty of Pharmacy, Zagazig University, Zagazig, Egypt; ^2^Department of Pharmacology & Toxicology, Faculty of Pharmacy, Tabuk University, Tabuk, Saudi Arabia

## Abstract

This study was designed to investigate the impact of oral administration of fenitrothion (10 mg/kg) on liver, kidney, brain, and lung function in rats. The effect was studied on days 7, 14, 21, 28, and 42. Our results have shown deterioration in liver function as evidenced by the elevation in serum ALT, AST, ALP, and bilirubin and reduction in albumin and hepatic glycogen. This was associated with a state of hyperglycemia and hyperlipidemia and increased prothrombin time, while hemoglobin content was reduced. In addition, the kidney function was reduced as indicated by the elevation in serum creatinine, uric acid, and BUN, while the serum levels of magnesium, potassium, and sodium were reduced. This study also showed an impairment in brain neurotransmitter (elevated 5-HT, glutamate, GABA, and reduced dopamine and norepinephrine level). This was associated with a reduction in the barrier capacity in brain and lung. Fenitrothion also caused a decrease in cholinesterase activity in serum, lung, and brain activity associated with a state of oxidative stress in all tested organs and hyperammonemia. These results support the hazards of pesticide use and shows the importance of minimizing pesticide use or discovering new safe pesticides.

## 1. Introduction

Organophosphorus pesticides (OPs) are among the most widely used insecticides globally and they are readily available commercially for domestic and industrial purposes [1]. The widespread use of OPs by public health and agricultural programs has led to severe environmental pollution [2, 3] that constitutes a significant potential health hazard because of the possibility of the acute or chronic poisoning of humans and animals [4].

Fenitrothion is one of the most widely used organophosphorus pesticides mainly used in agriculture for controlling chewing and sucking insects. It is also used for the control of flies, mosquitos, and cockroaches in public health programs and/or indoor use [5].

Organophosphates affect many vital organs; chronic toxicity with organophosphorus pesticides may cause extreme injury in liver cells [6]. Liver enzymes, endogenous antioxidant status, and essential trace elements were found to be adversely affected after chronic OPs intoxication to rats [7]. In addition, hematological parameters such as hemoglobin, leucocyte count, and coagulation of blood have been considered as bioindicators of toxicities following chronic exposure to malathion [8] and pyrethroids [9, 10].

Neuronal necrosis has been observed in multiple cortical and subcortical regions in experimental rats exposed to OPs [6] as soman [11, 12], fenthion [13], and methamidophos [14]. In addition, symptoms of chronic OPs toxicity vary between headache, sweating, Parkinson's, alterations in memory, and psychiatric or neuropsychological dysfunction [15, 16]. In addition, the key findings of OPs toxicity in respiratory system include shortness of breath and rapidly progressive bradypnea leading to apnea due to loss of central inspiratory drive causing central failure of breathing [17]. Chronic exposure to organophosphorus pesticides leads to kidney failure [18]. It has also been reported that pesticides exposure was associated with kidney cancer [1].

The present study was designed to evaluate the consequences of oral fenitrothion administration for 42 consecutive days on liver function and its possible deleterious action on brain, lung, and kidney in albino rats.

## 2. Materials and Methods

Fenitrothion (Sumithion 50®, 500 mg/mL) was purchased from Kafr Elzayat Co. for Insecticide Ind., (Kafr Elzayat, Egypt). Fenitrothion emulsion was freshly diluted in distilled water to 10 mg/mL and orally administered at a dose of 1 mL/kg rat body weight which corresponds to 10 mg/kg. The difference in administered volume among animals was not more than 12% based on body weight differences. The dose of fenitrothion was selected based on a previous study that used fenitrothion at 10 and 20 mg/kg [19].

### 2.1. Animals

Male albino rats weighing 160 ± 10 g were obtained from National Research Center (Cairo, Egypt) and were housed in plastic cages and allowed free access to a standard diet and tap water. The rats were housed at 23 ± 2°C 12 hr dark/light cycle. All experimental procedures were approved by the Ethical Committee for Animal Handling at Zagazig University (ECAHZU) (number P7-3-2013 and number P8-3-2013).

Animals were randomly allocated into 6 groups (*n* = 10) treated daily with the following: C (control group treated with oral distilled water, 1 mL/kg for 42 days), F1 (oral fenitrothion, 1 mL/kg for 7 days), F2 (oral fenitrothion, 1 mL/kg for 14 days), F3 (oral fenitrothion, 1 mL/kg for 21 days), F4 (oral fenitrothion, 1 mL/kg for 28 days), and F6 (oral fenitrothion, 1 mL/kg for 42 days).

At the end of the experiment, after overnight fasting, blood was collected from the retroorbital plexus and centrifuged at 3500 rpm for 15 minutes with or without heparin and serum/plasma was collected and stored at −20°C. Animals were sacrificed by decapitation and liver, brain cortex, lung, and kidney were excised for preparation of tissue homogenates. The cortex was chosen because the control centers of consciousness and memory are located mainly in cerebral cortex [20].

### 2.2. Methods

AChE activity in brain cortex and lung tissue was determined colorimetrically using acetylthiocholine iodide and 5,5′-dithiobis (nitrobenzoate) (DTNB) at 412 nm [21]; amino acids content was determined fluorometrically using ninhydrin at excitation of 377 nm and emission of 451 nm for both glutamate and GABA [22, 23], while content of monoamines was determined fluorometrically using n-heptane at excitation at 320 nm and emission at 480 nm for DA, excitation at 380 nm and emission at 480 nm for NE, and excitation at 355 nm and emission at 470 nm for 5-HT [23]. Water content in brain and lung tissue was determined using wet and dry weight method [24]. Evans blue extravasation in brain and lung tissue was determined colorimetrically using Evans blue dye at 610 nm [24, 25].

Liver glycogen content was determined by the gravimetric method using KoH, trichloroacetic acid, and absolute ethanol [26] and catalase (CAT) activity in tissue (liver, brain, lung, and kidney) was determined colorimetrically using potassium dichromate at 570 nm, reduced glutathione (GSH) content in tissue (liver, brain, lung, and kidney) was determined colorimetrically using 5,5′-dithiobis (nitrobenzoate) (DTNB) at 412 nm, and malondialdehyde (MDA) content in tissue (liver, brain, lung, and kidney) was determined colorimetrically using thiobarbituric acid (TBA) and trichloroacetic acid at 535 nm [27–29].

The following parameters were assayed in serum using kits supplied by Biodiagnostic Co. (Cairo, Egypt): ALT and AST [30], ALP [31], butyrylcholinesterase activity [32], total bilirubin [33], albumin [34], sodium [35], potassium [36], magnesium [37], cholesterol [38], HDL-cholesterol [39], LDL-cholesterol [40], blood urea nitrogen (BUN) [41], creatinine, and uric acid contents [42].

Hemoglobin content [43], glucose level [44], and prothrombin time [45] were determined in blood using kits supplied by Biodiagnostic Co. (Cairo, Egypt).

Liver, serum, brain, lung, and kidney total protein content were determined by colorimetric method using a kit supplied by Biodiagnostic Co. (Cairo, Egypt) [46].

Liver, serum, and brain ammonia content were determined by colorimetric method using a kit supplied by Biodiagnostic Co. (Cairo, Egypt) [47].

### 2.3. Statistical Analysis

Data are expressed as means ± SD. The statistical significance of the data was determined using one-way analysis of variance (ANOVA) followed by Tukey's post hoc test using SPSS software package version 10. The level of significance was taken as *p* < 0.05.

## 3. Results

### 3.1. Effect on Some Liver Functions

**Figure 1:**
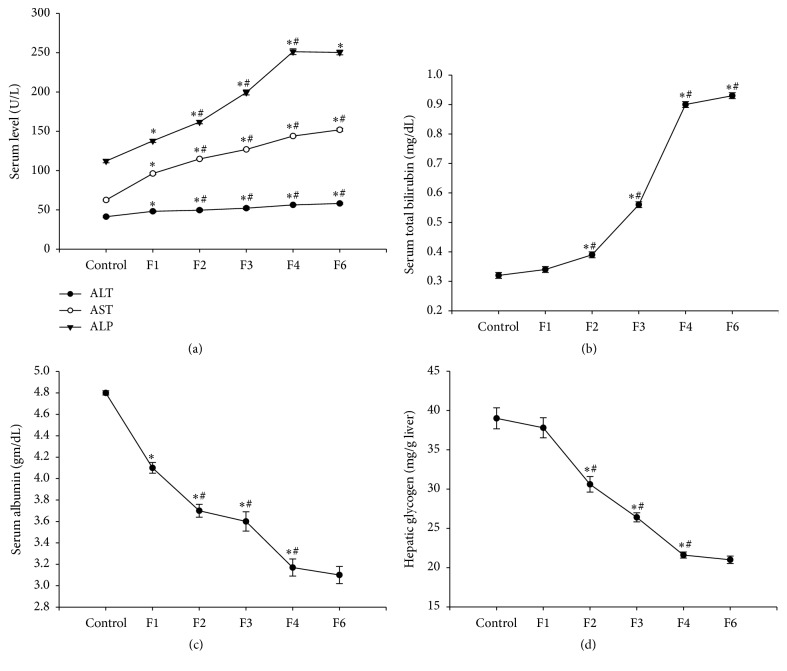
Effects of fenitrothion (10 mg/kg) for 1, 2, 3, 4, and 6 weeks (F1, F2, F3, F4, and F6, resp.) on some liver functions in rats. Data are expressed as mean ± SD. *N* = 10. ^*∗*^Significantly different from control group; ^#^significantly different from fenitrothion at the previous time point at *p* < 0.05 using ANOVA followed by LSD and Tukey's post hoc test.

Administration of fenitrothion for 7, 14, 21, 28, and 42 days (F1, F2, F3, F4, and F6, resp.) caused a significant gradual increase in serum ALT activity compared with control group (48, 49.6, 52.1, 56.3, and 58.1 versus 41.3 u/l), AST activity compared with control group (96.2, 114.8, 126.9, 143.9, and 151.9 versus 62.5 u/l), ALP activity compared with control group (137.8, 161.8, 199.4, 251.3, and 250.3 versus 112.1 u/l) (Figure 1(a)), and serum total bilirubin level compared with control group (0.56, 0.9, and 0.93 versus 0.32 mg/dL) (Figure 1(b)).

On the other hand, administration of fenitrothion for 7, 14, 21, 28, and 42 days (F1, F2, F3, F4, and F6, resp.) caused a significant gradual decrease in serum albumin compared with control group (4.11, 3.73, 3.59, 3.17, and 3.1 versus 4.89 g/dL) (Figure 1(c)) and liver glycogen content compared with control group (3.06, 2.64, 2.16, and 2.1 versus 3.9 g/100 g liver) (Figure 1(d)).

### 3.2. Effects on Liver Histopathology

**Figure 2:**
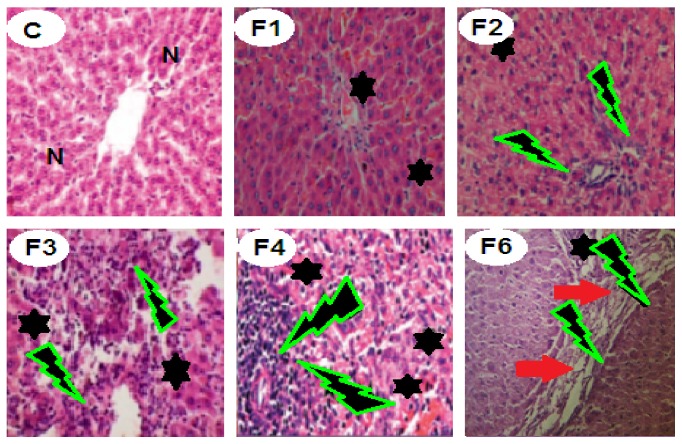
Effects of fenitrothion on liver histopathology in rats using H & E staining (×400). C: normal hepatic parenchyma, F1: mild congested, disorganized hepatic lobules and slight cell swelling, F2: moderate cell swelling associated with apoptosis and focal necrosis, F3: moderate degenerated and ballooned necrotic hepatocytes with lymphocytic aggregation and cell infiltration, F4: severe disappearance of hepatocytes and large areas of congestion, lymphocytic aggregation, and inflammation, and F6: thickened interlobular tissue by edematous fibrous tissue and congested blood vessels. N: normal liver cells. Black star: congested cells with cytoplasmic vacuolation. Green lightning: lymphocytic aggregation. Red arrow: edematous fibrous tissue.

Administration of fenitrothion for 7, 14, 21, 28, and 42 days (F1, F2, F3, F4, and F6, resp.) caused significant gradual deterioration in liver tissue starting by disorganized lobular patterns in hepatic parenchyma, followed by congested hepatic tissue and programmed cell death (apoptosis) and focal necrosis and widening of the hepatic sinusoids beside cell swelling in the remaining hepatic parenchyma, and ending with interstitial lymphocytic aggregations and interlobular thickened edematous fibrous tissue (Figure 2).

### 3.3. Effect on Some Hematologic Parameters

**Figure 3:**
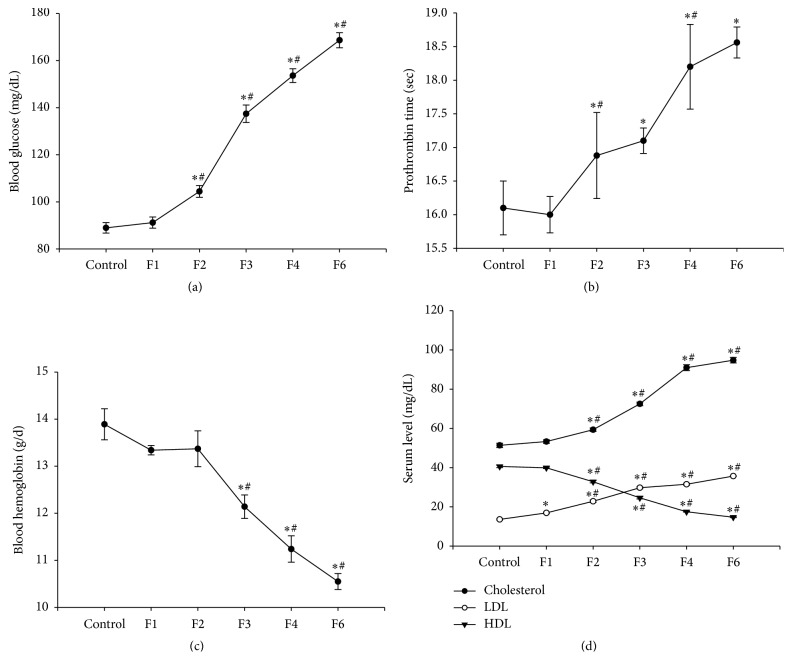
Effects of fenitrothion (10 mg/kg) for 1, 2, 3, 4, and 6 weeks (F1, F2, F3, F4, and F6, resp.) on some blood parameters in rats. Data are expressed as mean ± SD. *N* = 10. ^*∗*^Significantly different from control group; ^#^significantly different from fenitrothion at the previous time point at *p* < 0.05 using ANOVA followed by Tukey's post hoc test.

Administration of fenitrothion for 14, 21, 28, and 42 days (F2, F3, F4, and F6, resp.) showed a significant gradual increase in blood glucose level compared with control group (104.43, 137.41, 153.63, and 168.66 versus 88.96 mg/dL) (Figure 3(a)) and prothrombin time compared with control group (16.88, 17.12, 18.22, and 18.56 versus 16.12 sec.) (Figure 3(b)).

This was accompanied by a significant decrease in blood hemoglobin content (by 12, 19, and 24%, resp.) compared with control group starting from week 3 of fenitrothion administration (F3) that continued throughout the experiment (F4 and F6) (Figure 3(c)).

Administration of fenitrothion for 14, 21, 28, and 42 days (F2, F3, F4, and F6, resp.) showed a state of hyperlipidemia as evidenced by the significant gradual elevation in serum cholesterol compared with control group (59.33, 72.49, 90.96, and 94.76 versus 51.35 mg/dL) and LDL content compared with control group (16.9, 22.8, 29.7, 31.51, and 35.67 versus 13.61 mg/dL) and the reduction in serum HDL compared with control group (32.7, 24.6, 17.2, and 14.7 versus 40.6 mg/dL) compared with control group (Figure 3(d)).

### 3.4. Effect on Kidney Function

**Figure 4:**
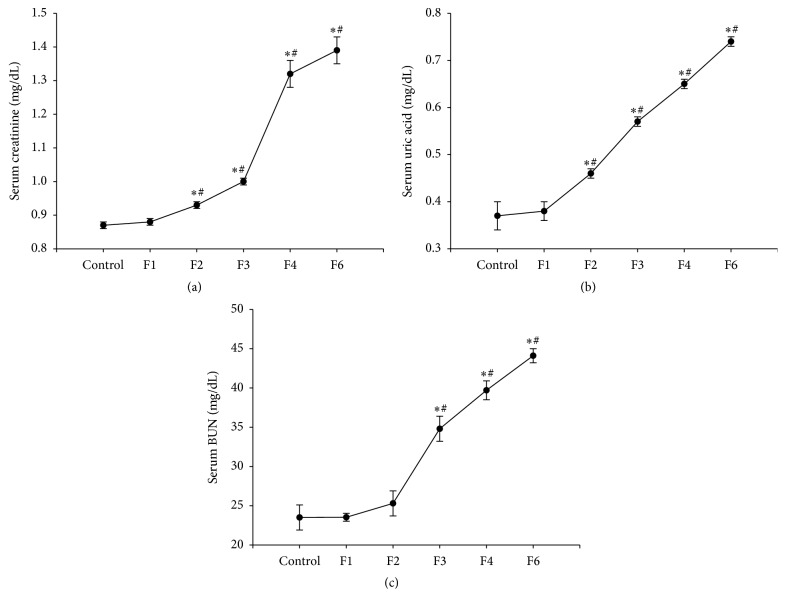
Effects of fenitrothion (10 mg/kg) for 1, 2, 3, 4, and 6 weeks (F1, F2, F3, F4, and F6, resp.) on some kidney functions in rats. Data are expressed as mean ± SD. *N* = 10. ^*∗*^Significantly different from control group; ^#^significantly different from fenitrothion at the previous time point at *p* < 0.05 using ANOVA followed by Tukey's post hoc test.

Administration of fenitrothion for 14, 21, 28, and 42 days (F2, F3, F4, and F6, resp.) caused a significant gradual increase in serum creatinine content compared with control group (0.88, 0.93, 1.01, 1.32, and 1.39 versus 0.87 mg/dL) (Figure 4(a)) and uric acid (Figure 4(b)), while serum blood urea nitrogen (BUN) content compared with control group (24, 25, 35, 40, and 44 versus 24 mg/dL) was increased starting from F3 (Figure 4(c)).

### 3.5. Effects on Kidney Histopathology

**Figure 5:**
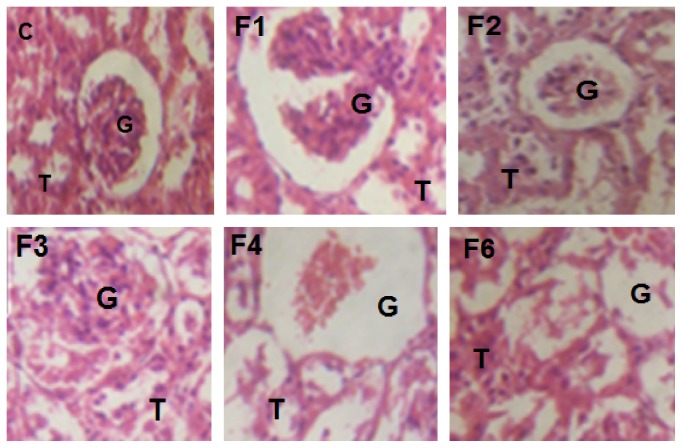
Effects of fenitrothion on kidney histopathology in rats using H & E staining (×400). C: normal segments of nephrons, interstitium, and blood vessels, F1: degenerative changes in glomeruli and tubules, F2: partial necrosis of glomerular tafts and some renal tubular epithelium in renal cortex, F3: focal intense degenerative changes in renal tubules and few shrunken glomeruli, F4: diffuse necrotic changes in renal tubules and glomeruli, and F6: marked necrosis of tubular cells, atrophy of the glomeruli, and areas of interstitial infiltration of round cells. G: renal glomeruli and T: renal tubule.

Administration of fenitrothion for 7, 14, 21, 28, and 42 days (F1, F2, F3, F4, and F6, resp.) caused a gradual significant histopathological changes in kidney tissue starting from minor changes in renal tubules as well as glomeruli at the cortical portion and ending with severe degenerative changes in kidney tissue compared with control group (Figure 5).

### 3.6. Effect on Electrolyte

**Figure 6:**
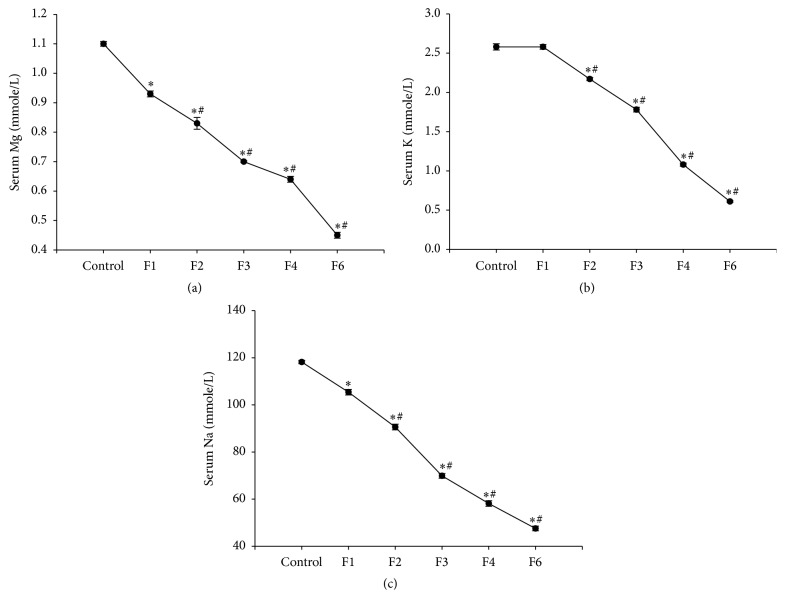
Effects of fenitrothion (10 mg/kg) for 1, 2, 3, 4, and 6 weeks (F1, F2, F3, F4, and F6, resp.) on serum electrolytes in rats. Data are expressed as mean ± SD. *N* = 10. ^*∗*^Significantly different from control group; ^#^significantly different from fenitrothion at the previous time point at *p* < 0.05 using ANOVA followed by Tukey's post hoc test.

Administration of fenitrothion for 7, 14, 21, 28, and 42 days (F1, F2, F3, F4, and F6, resp.) induced a significant gradual reduction in serum magnesium content compared with control group (0.93, 0.83, 0.7, 0.64, and 0.45 versus 1.1 mmol/l), potassium content compared with control group (2.17, 1.78, 1.08, and 0.61 versus 2.62 mmol/l), and sodium content compared with control group (105.4, 90.6, 69.9, 58.2, and 47.6 versus 118.2 mmol/l) (Figures 6(a), 6(b), and 6(c)).

### 3.7. Effect on Brain Neurotransmitters

**Figure 7:**
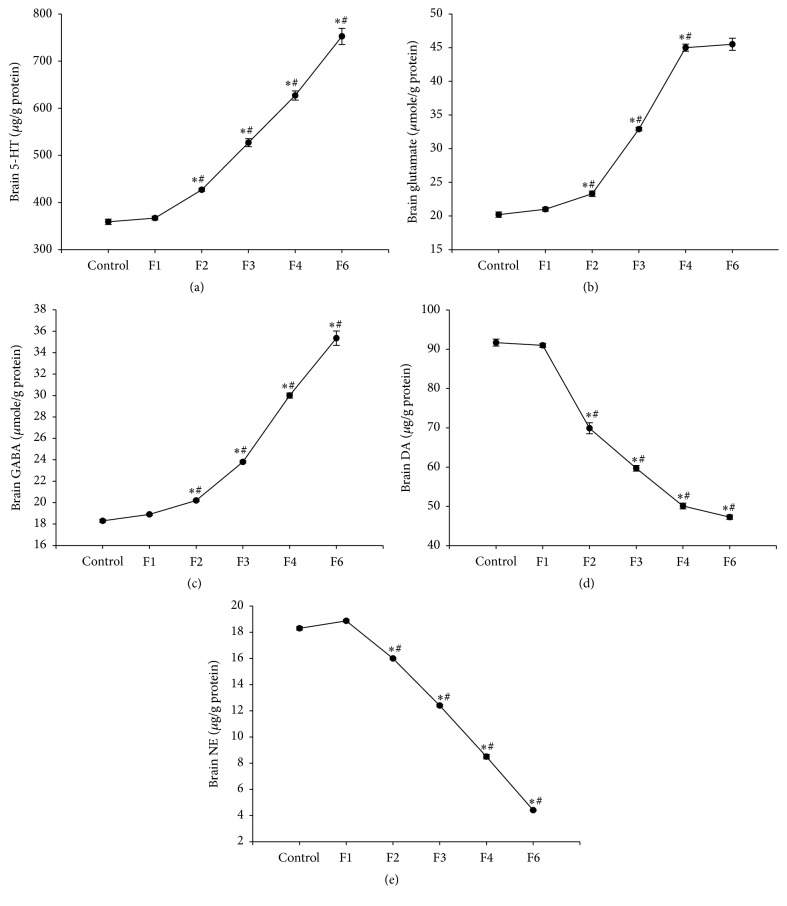
Effects of fenitrothion (10 mg/kg) for 1, 2, 3, 4, and 6 weeks (F1, F2, F3, F4, and F6, resp.) on some brain neurotransmitters in rats. Data are expressed as mean ± SD. *N* = 10. ^*∗*^Significantly different from control group; ^#^significantly different from fenitrothion at the previous time point at *p* < 0.05 using ANOVA followed by Tukey's post hoc test.

Administration of fenitrothion for 14, 21, 28, and 42 days (F2, F3, F4 and F6, resp.) showed a significant gradual increase in brain 5-HT content compared with control group (427.88, 527.89, 627.38, and 752.46 versus 359.21 *μ*g/g protein), glutamate content compared with control group (23.37, 32.9, 45.09, and 46.46 versus 20.24 *μ*mol/g protein), and GABA content in cortex compared with control group (20.18, 23.84, 30.5, and 35.35 versus 18.34 *μ*mol/g protein) (Figures 7(a), 7(b), and 7(c)).

On the other hand, administration of fenitrothion for 14, 21, 28, and 42 days (F2, F3, F4, and F6, resp.) was associated with a significant gradual reduction in brain DA content compared with control group (69.99, 59.71, 50.09, and 47.27 versus 91.72 *μ*g/g protein) and NE content in brain cortex compared with control group (16, 12.42, 8.53, and 4.41 versus 18.34 *μ*g/g protein) (Figures 7(d) and 7(e), resp.).

### 3.8. Effect on Brain and Lung Barrier Integrity

**Figure 8:**
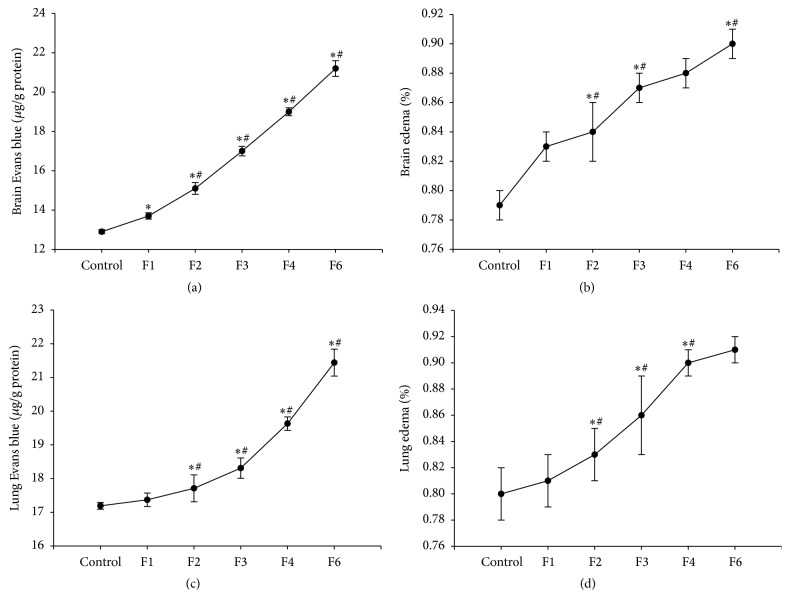
Effects of fenitrothion (10 mg/kg) for 1, 2, 3, 4, and 6 weeks (F1, F2, F3, F4, and F6, resp.) on barrier integrity in rats. Data are expressed as mean ± SD. *N* = 10. ^*∗*^Significantly different from control group; ^#^significantly different from fenitrothion at the previous time point at *p* < 0.05 using ANOVA followed by Tukey's post hoc test.

Administration of fenitrothion for 7, 14, 21, 28, and 42 days (F1, F2, F3, F4, and F6, resp.), revealed a significant gradual decrease in blood brain barrier integrity as evidenced by the increase in Evans blue extravasation in brain compared with control group (13.7, 15.14, 17.1, 19.06, and 21.42 versus 12.91 *μ*g/g protein) (Figure 8(a)). This was accompanied by a significant increase in brain water content compared with control group (0.84, 0.87, 0.88, and 0.91 versus 0.79 g) starting from F2 (Figure 8(b)).

Similarly, administration of fenitrothion for 14, 21, 28, and 42 days (F2, F3, F4, and F6, resp.) revealed a significant gradual decrease in lung barrier integrity as evidenced by the increase in Evans blue extravasation in lung compared with control group (18.31, 19.63, and 21.44 versus 17.19 *μ*g/g protein) (Figure 8(c)) which was also accompanied by a significant increase in lung water content compared with control group (0.86, 0.9, and 0.91 versus 0.8 g) starting from F2 (Figure 8(d)).

### 3.9. Effects on Brain Histopathology

**Figure 9:**
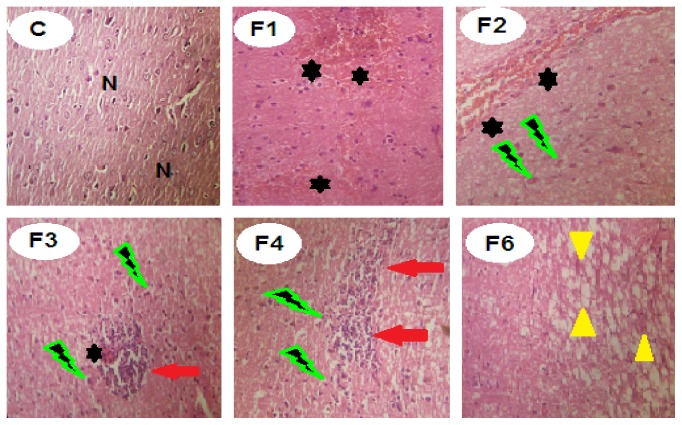
Effects of fenitrothion on brain histopathology in rats using H & E staining (×400). C: normal brain tissue, F1: focal hemorrhagic areas in the cortex, F2: scattered hemorrhage in brain ventricles and cytotoxic edema, F3: focal hemorrhagic areas, cytotoxic edema, degenerated neurons, and focal microgliosis, F4: cytotoxic edema and intense microgliosis, and F6: degenerated neurons, demyelination of the white matter axons, and encephalomalacia. N: normal brain cells. Black star: hemorrhagic area. Green lightning: cytotoxic edema. Red arrow: degenerated neurons and focal microgliosis. Yellow head arrow: demyelination of the white matter axons and encephalomalacia.

Administration of fenitrothion for 7, 14, 21, 28, and 42 days (F1, F2, F3, F4, and F6, resp.) caused significant gradual deterioration in brain tissue starting by focal scattered hemorrhagic areas. Later on, intense haemorrhage and narrow spaces with early encephalomalacia became visible in both lateral ventricles. This was followed by demyelination of nerve axon in the white matter, encephalomalacia, cytotoxic edema, neuronal degeneration, and intense microgliosis (Figure 9).

### 3.10. Effects on Lung Histopathology

**Figure 10:**
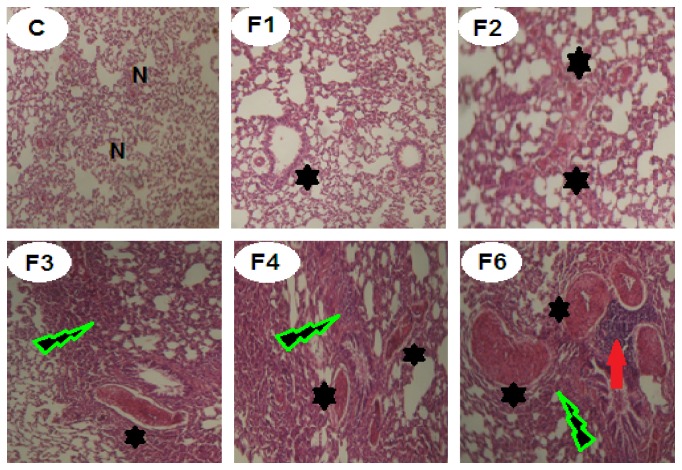
Effects of fenitrothion on lung histopathology in rats using H & E staining (×400). C: normal lung tissue, F1: slight hypertrophied vascular smooth muscles, F2: mild hypertrophied vascular smooth muscles, F3: moderate hypertrophied vascular smooth muscles and thickened intra-alveolar septa, F4: severe hypertrophied vascular smooth muscles and thickened intra-alveolar septa, and F6: severe hypertrophied vascular smooth muscles, interstitial hemorrhages with narrow alveolar spaces, and intense thickened septa with perivascular leukocytic aggregation. N: normal lung cells. Black star: hypertrophied vascular smooth muscles. Green lightning: thickening in the alveolar septa. Red arrow: perivascular leukocytic aggregation.

Administration of fenitrothion for 7, 14, 21, 28, and 42 days (F1, F2, F3, F4, and F6, resp.), caused significant gradual deterioration in the histopathology of lung tissue starting by focal hypertrophied smooth muscles of the pulmonary blood vessels and thickened intra-alveolar septa and ending with prevalent hypertrophied smooth muscles, interstitial hemorrhages with narrow alveolar spaces, and intense thickened septa with perivascular leukocytic aggregation compared to control group (Figure 10).

### 3.11. Effects on Cholinesterase Activity and Ammonia Content

**Figure 11:**
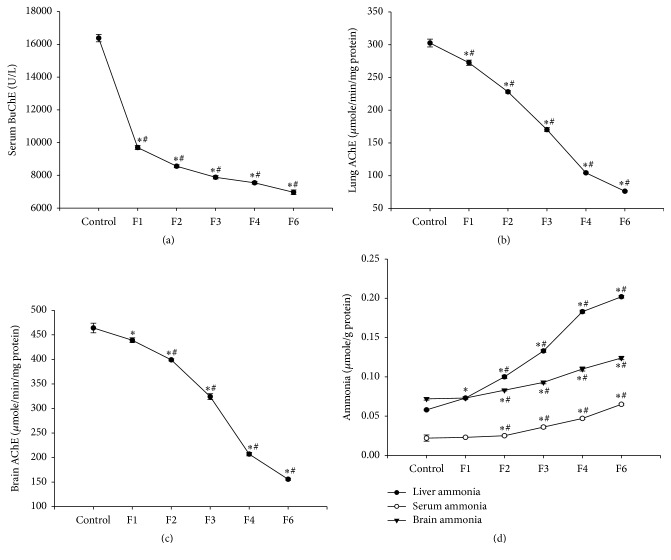
Effects of fenitrothion (10 mg/kg) for 1, 2, 3, 4, and 6 weeks (F1, F2, F3, F4, and F6, resp.) on cholinesterase activity and ammonia level in rats. Data are expressed as mean ± SD. *N* = 10. ^*∗*^Significantly different from control group; ^#^significantly different from fenitrothion at the previous time point at *p* < 0.05 using ANOVA followed by Tukey's post hoc test.

Administration of fenitrothion for 7, 14, 21, 28, and 42 days (F1, F2, F3, F4, and F6, resp.) caused a significant gradual decrease in serum butyrylcholinesterase activity compared with control group (9696.3, 8557.64, 7879.32, 7544.74, and 6963.54 versus 16380 u/l), lung acetylcholinesterase activity compared with control group (272.41, 228.03, 170.32, 104.28, and 76.16 versus 302.49 *μ*mol/min/mg protein), and brain acetylcholinesterase compared with control group (439.68, 399.16, 324.57, 207.26, and 155.76 versus 464.93 *μ*mol/min/mg protein) (Figures 11(a), 11(b) and 11(c)).

On the other hand, fenitrothion caused a significant increase in liver ammonia content compared with control group (0.073, 0.1, 0.133, 0.183, and 0.202 versus 0.058 *μ*mol/g protein) starting from the first week of administration (F1) and continuing throughout the experiment (F2, F3, F4, and F6). In addition, fenitrothion caused a significant increase in ammonia content in serum compared with control group (0.025, 0.036, 0.047, and 0.065 versus 0.022 *μ*mol/l) and brain ammonia content compared with control group (0.083, 0.093, 0.11, and 0.124 versus 0.072 *μ*mol/g protein) starting from week 2 of fenitrothion administration (F2) and continuing throughout the experiment (F3, F4, and F6) as shown in Figure 11(d).

### 3.12. Effect on Oxidative Stress Biomarkers

**Figure 12:**
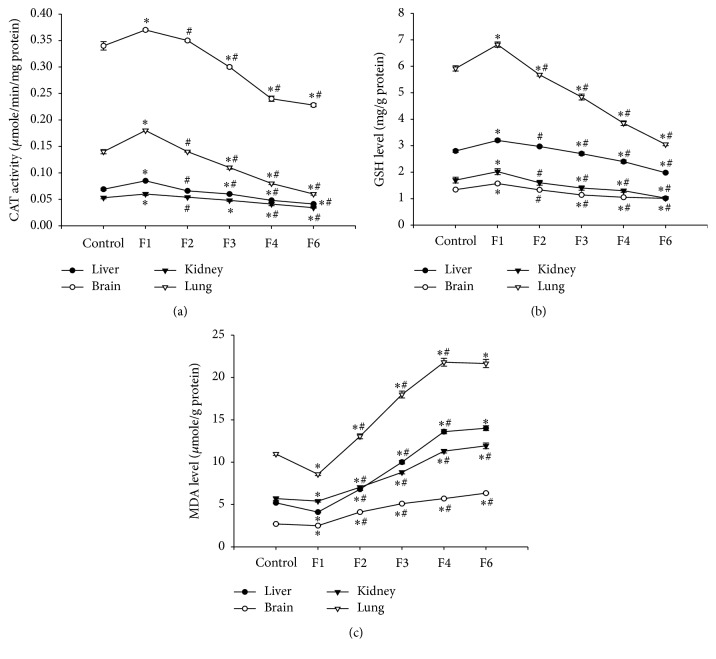
Effects of fenitrothion (10 mg/kg) for 1, 2, 3, 4, and 6 weeks (F1, F2, F3, F4, and F6, resp.) on antioxidant state in rats. Data are expressed as mean ± SD. *N* = 10. ^*∗*^Significantly different from control group. ^#^Significantly different from fenitrothion at the previous time point at *p* < 0.05 using ANOVA followed by Tukey's post hoc test.

Administration of fenitrothion caused a significant initial increase in catalase (CAT) activity after 7 days (F1) in liver, brain, kidney, and lung compared with control group (0.085 versus 0.069, 0.37 versus 0.34, 0.06 versus 0.053, and 0.18 versus 0.14 *μ*mole/min/mg, resp.). However, continued administration of fenitrothion caused a gradual significant decrease in CAT activity in liver, brain, kidney, and lung after 21, 28, and 42 days (F3, F4, and F6, resp.) compared with control group (0.06, 0.048, and 0.041 versus 0.069 in liver, 0.3, 0.24, and 0.22 versus 0.34 in brain, 0.048, 0.041, and 0.034 versus 0.053 in kidney, and 0.11, 0.08, and 0.06 versus 0.14 *µ*mole/min/mg in lung) (Figure 12(a)).

Similarly, fenitrothion caused a significant initial increase in glutathione content (GSH) after 7 days (F1) in liver, brain, kidney, and lung compared with control group (3.2 versus 2.8, 1.57 versus 1.34, 2.02 versus 1.7, and 6.8 versus 5.9 mg/g, resp.). This was followed by a gradual significant decrease in GSH content in liver, brain, kidney, and lung after 21, 28, and 42 days (F3, F4, and F6, resp.) compared with control group (2.7, 2.4, and 1.98 versus 2.8 in liver, 1.14, 1.05, and 1.01 versus 1.34 in brain, 1.4, 1.3, and 1.02 versus 1.7 in kidney, and 4.84, 3.85, and 3.05 versus 5.92 mg/g in lung) (Figure 12(b)).

On the other hand, administration of fenitrothion for 7 days (F1) caused a significant decrease in malondialdehyde (MDA) content in liver, brain, lung, and kidney compared with control group (4.1 versus 5.2, 2.5 versus 2.7, 5.4 versus 5.7, and 8.57 versus 10.96 *μ*mole/g, resp.). Continued administration of fenitrothion caused a gradual significant decrease in MDA content in liver, brain, kidney, and lung after 14, 21, 28, and 42 days (F2, F3, F4, and F6, resp.) compared with control group (6.8, 10, 13.6, and 14 versus 5.2 in liver, 4.1, 5.1, 5.7, and 6.34 versus 2.7 in brain, 7.04, 8.8, 11.3, and 11.93 versus 5.7 in kidney, and 13.05, 17.99, 21.8, and 21.65 versus 10.96 *μ*mole/g in lung) (Figure 12(c)).

## 4. Discussion

This study was designed to investigate the effect of the organophosphorus pesticide: fenitrothion (10 mg/kg/day, p.o. for 6 weeks) on vital organs (liver, blood, kidney, brain, and lung) in albino rats.

Concerning the effect of fenitrothion on hepatocellular integrity, this study showed significant gradual increase in ALT and AST activity compared to control group starting from the first week. These results coincide with previous studies that showed a significant increase in liver enzymes in rats exposed to organophosphorus insecticides as fenitrothion [48].

The alteration in these enzymes might be attributed to hepatocellular damage and alteration in permeability of cell membrane leading to leakage of these enzymes into blood stream [49].

In the present study, fenitrothion administration gradually increased ALP activity as compared to control group. This result is in agreement with Kalender at al. [1], who reported that malathion treated rats had significantly higher ALP activity than the control group. This change may be in response to enhanced dephosphorylation of ALP for further metabolism of fenitrothion to be excreted with bile or due to defect in ALP excretion in bile by hepatocytes [50].

In the present study, fenitrothion treated rats showed significant gradual increase in the level of bilirubin which is a normal metabolic product of hemoglobin in red blood cells [51] as previously shown by Jayusman et al. [5] who reported that ingestion of pesticides caused a significant increase in bilirubin level.

Additionally, hyperbilirubinemia is toxic to the central nervous system and may lead to a sequence of neurological symptoms and signs termed bilirubin encephalopathy [52].

The present study showed a gradual decrease in albumin content which coincides with [53] who showed reduction in albumin content after OPs exposure confirming the failure in liver functions [54]. The reduction in albumin content may reflect increased albumin metabolism [55] or decreased hepatic capacity to synthesize proteins due to increased number of injured hepatocytes [56].

The present study showed that fenitrothion administration caused deterioration in glucose homeostasis as evidenced by significant gradual increase in blood glucose level and a drop in hepatic glycogen content; a previous study have reported that chronic exposure to OPs participates in hyperglycemia and depletion of glycogen content in liver and muscle [57, 58].

This might be attributed to disturbed glucose transport and glycogen metabolism [59]. Additionally, increased blood glucose level may result from an imbalance between hepatic output of glucose and its peripheral uptake [60] or as a consequence of abruptly increased catabolism to meet higher OP induced energy demands [61].

In addition, fenitrothion caused a significant gradual increase in prothrombin time starting from week 2. Reference [53] showed alteration in coagulation after OPs exposure confirming the failure in liver functions and decreased hepatic capacity to synthesize coagulation factors [56].

The current study showed that exposure to fenitrothion was accompanied by a significant gradual decrease in the level of hemoglobin from the first week which might explain the state of hyperbilirubinemia since bilirubin is a normal metabolic product of hemoglobin in red blood cells [51]. These results are in accordance with those obtained by [62], who reported that OPs exposure altered hematological parameters in rats.

The reduction in hemoglobin level might be attributed to impairment of biosynthesis of heme in bone marrow [63] or binding of OPs on iron, followed by a lack of incorporation of iron in hemoglobin [64].

The current study has shown a gradual increase in serum cholesterol and LDL and gradual decrease in HDL after administration of fenitrothion. These results are in accordance with those obtained by Kalender at al. [1]. Liver is involved in lipid synthesis, metabolism, and transportation and changes in plasma lipid level could serve as a simple marker for assessing liver disorders [65]. These alterations in lipid profile might be attributed to the effect of fenitrothion on the permeability of hepatocyte cell membrane or blockage of the bile duct which reduces or stops cholesterol secretion into the duodenum [1]. Also, this could be attributed to increased hepatic synthesis and/or diminished hepatic degradation of lipids due to reduced lipoprotein lipase activity [66].

This study showed that fenitrothion caused a gradual elevation in BUN, serum creatinine, and serum uric acid starting from third week indicating functional damage of the kidney. These results coincide with previous studies which demonstrated that the kidney is one of the target organs of OPs [67, 68].

In this study, fenitrothion caused a gradual decrease in serum Mg^++^, K^+^, and Na^+^ content. The present findings are consistent with [69] that reported that exposure to pesticides caused decrease in serum Mg, K, and Na level. The importance of serum electrolytes is correlated with their involvement in many vital activities including muscle contraction, maintenance of acid-base balance, and nerve impulse conduction [70].

Gumz et al. [71] correlated these changes with alteration of the membrane configuration accompanied by cell membrane damage and disorders in membrane permeability leading to alteration in electrolyte balance.

Additionally, Mossalam et al. [72] showed that lipid peroxidation products generated by pesticides can cause DNA damage and directly inhibit protein synthesis including Na^+^/K^+^ ATPase.

Hypokalemia can exacerbate hepatic encephalopathy by increasing renal ammoniagenesis and hence increasing systemic ammonia level [73]. Furthermore, Giuliani and Peri [74] reported that, during hyponatremia, excess water enters the brain and the cells get swollen producing hyponatremic encephalopathy.

In the present study, fenitrothion caused a gradual increase in brain glutamate, GABA, and serotonin (5-HT) content, while the contents of norepinephrine and dopamine were gradually decreased. Rivera-Espinosa et al. [75] have previously reported similar changes of brain neurotransmitters after liver injury. Additionally, Ahmed et al. [76] have previously reported similar changes of brain neurotransmitters after OPs exposure. Additionally, exposures to pesticides have been reported to cause permanent alterations in GABAergic, serotonergic [77], and dopaminergic systems [78].

The changes observed in GABAergic tone might be due to modulation of central and peripheral benzodiazepine receptors [79], while changes in glutamatergic, dopaminergic, and cholinergic system were attributed to disturbances in the synthesis and degradation of amino acids by the liver [80] and amino acid efflux from and/or reuptake by astrocytes [81].

In the present study, a gradual increase in Evans blue extravasation in brain tissue and brain water content was observed following fenitrothion administration. Like other OPs, fenitrothion can alter and cross the BBB, leading to neurological damage [82]. With the alteration in BBB permeability, brain water and sodium content are increased causing brain edema as previously reported [82]. In addition, metabolism of ammonia in astrocytes leads to glutamine accumulation that may contribute to astrocyte swelling, cytotoxic brain edema impaired astrocyte/neuronal communication, and synaptic plasticity [83].

In a similar manner, a gradual increase in EBE in lung tissue and lung water content was observed after fenitrothion administration. OPs can alter and cross the pleural membrane and cause lung injury through different mechanisms including ROS generation leading to pulmonary dysfunction. With the increase in pleural permeability of pulmonary microvessels, lung water content was increased profoundly causing lung edema [84].

Additionally, fluid accumulation causing brain and lung edema may also be due to the decreased production of albumin by the liver. As albumin production diminishes, fluid begins to leak from vessels causing edema that can lead to alteration of mental status and increasing in breathing rate and effort [85].

Organophosphorus pesticides have been reported to induce toxicity in mammals by inhibiting butyrylcholinesterase activity (BuChE) [86] and acetylcholinesterase (AChE), which leads to the accumulation of acetylcholine and the subsequent activation of cholinergic muscarinic and nicotinic receptors [87], leading to neuronal excitation and then paralysis of cholinergic transmission [88]. Therefore, acetylcholinesterase (AChE) and butyrylcholinesterase (BuChE) inhibition are a well-accepted index of organophosphorus insecticides intoxication [89].

The present study has demonstrated a gradual decline in serum BuChE and brain and lung AChE activity starting from first week of fenitrothion administration. Cholinesterases inhibition may be due to direct effect of fenitrothion [90] or indirect effect of hyperammonemia and/or decreased synthetic function of the liver as a result to hepatopathy [91].

Ammonia is normally converted to urea in the liver by a series of enzymatic reactions [92]. One important finding of this study was the gradual elevation in liver ammonia starting from the first week of fenitrothion administration while a significant gradual increase in plasma and brain ammonia started from the second week.

These results coincide with those reported by Scott et al. [93], who showed a significant increase in plasma ammonia following liver injury in rats, and Penchalamma and Jacop [94] who reported the elevation in ammonia level in liver, blood, and kidney after OPs exposure.

The elevated serum ammonia content can cross the blood brain barrier (BBB) [95] causing hepatic encephalopathy through several mechanisms, including impaired brain energy metabolism and autoregulation of blood flow [79], free radical production, changes in lipid composition in brain due to alteration in lipid profile, increased BBB permeability, and brain edema [93].

In normal subjects, intestinal ammonia, produced from nitrogen products, is taken up by the liver and metabolized to urea. Liver disease, whether acute or chronic, is usually accompanied by diminished hepatic urea synthesis, depriving the body of its main route of ammonia detoxification [96].

Ammonia was shown to cause imbalance between excitatory and inhibitory neurotransmitters [79]. Therefore, ammonia content is widely used in the diagnosis of hepatic encephalopathy in cirrhotic patients with altered mental status [97].

The current study has shown that administration of fenitrothion resulted in an initial increase in antioxidant defense mechanisms as evidenced by initial increase in catalase activity and glutathione content and an initial decrease in malondialdehyde content in the first week which was reversed with continued administration of fenitrothion culminating in a state of oxidative stress in the liver, brain, lung, and kidney.

This indicates an initial attempt of the body to combat the oxidative stress state induced by fenitrothion which failed and resulted in an evident state of oxidative stress in liver, brain, and lung. Organophosphorus pesticides have been reported to cause oxidative stress and changes in antioxidant status system [98, 99]. Similar effects of OPs were previously reported by [1] in liver, [100] in brain, [101] in lung, and [67] in kidney.

Organophosphorus pesticide may induce oxidative stress through their “redox-cycling” activity, where they generate superoxide anions and hydrogen peroxide, or through ROS generation via changes in normal antioxidant homeostasis that results in depletion of antioxidants [102].

Organophosphorus pesticides have been shown to induce inflammation and cell infiltration [103].

In the present study the altered histopathological features of liver, brain, and lung were consistent with the biochemical changes and authenticated the injury caused by fenitrothion. Fenitrothion administration caused liver cell injury and congestion of some hepatic cells (in 1st week), apoptosis, focal necrosis and hepatic cell swelling (in 2nd week), ballooned necrotic hepatocytes with lymphocytic aggregation and cell infiltration (in 3rd week), large areas of congestion, lymphocytic aggregation and inflammation (in 4th week), interlobular tissue thickened by edematous fibrous tissue, and congested blood vessels (in 6th week). These changes are in line with data recorded by [104, 105].

Additionally, marked histopathological alterations in brain tissue were observed following fenitrothion administration including focal scattered hemorrhagic areas in cortex (in 1st week), ischemic changes in brain neurons and cytotoxic edema (in 2nd week), cytotoxic edema, degenerated neurons, and focal microgliosis (in 3rd week), cytotoxic edema and intense microgliosis (in 4th week), and finally degenerated neurons, demyelination of the white matter axons and encephalomalacia (in 6th week). Similar alterations were previously reported in chlorpyrifos (OPs) intoxicated animals [106].

Furthermore, marked histopathological alterations were observed in the lung of fenitrothion treated groups including slight hypertrophied vascular smooth muscles (in 1st week), mild hypertrophied vascular smooth muscles (in 2nd week), moderate hypertrophied vascular smooth muscles and thickened intra-alveolar septa (in 3rd week), severe hypertrophied vascular smooth muscles and thickened intra-alveolar septa (in 4th week), and severe hypertrophied vascular smooth muscles, interstitial hemorrhages with narrow alveolar spaces and intense thickened septa with perivascular leukocytic aggregation (in 6th week). These findings are supported by previous studies [84, 107].

## 5. Conclusion

In conclusion, these results demonstrated that intoxication with fenitrothion induced significant damage of the liver, brain, lung, and kidney leading to imbalance in liver, brain, lung, and kidney functions. Interestingly, the liver was the first to be affected by fenitrothion administration which might imply a delayed effect of fenitrothion on the kidney, brain, and lung due to pharmacokinetic influences or that the damaged liver has affected other organs through elevated ammonia, bilirubin, or defects in hepatic synthesis of vital proteins as albumin and clotting factors.

These results support the hazards of pesticide use and shows the importance of minimizing pesticide use or discovering new safe pesticides.

## References

[1] Kalender S., Uzun F. G., Durak D., Demir F., Kalender Y. (2010). Malathion-induced hepatotoxicity in rats: the effects of vitamins C and E. *Food and Chemical Toxicology*.

[2] Lasram M. M., Annabi A. B., Elj N. E. (2009). Metabolic disorders of acute exposure to malathion in adult Wistar rats. *Journal of Hazardous Materials*.

[3] Rekha R., Raina S., Hamid S. (2013). Histopathological effects of pesticide-cholopyrifos on kidney in albino rats. *International Journal of Research in Medical Sciences*.

[4] Hernández A. F., Parrón T., Tsatsakis A. M., Requena M., Alarcón R., López-Guarnido O. (2013). Toxic effects of pesticide mixtures at a molecular level: their relevance to human health. *Toxicology*.

[5] Jayusman P. A., Budin S. B., Ghazali A. R., Taib I. S., Louis S. R. (2014). Effects of palm oil tocotrienol-rich fraction on biochemical and morphological alterations of liver in fenitrothion-treated rats. *Pakistan Journal of Pharmaceutical Sciences*.

[6] Kumar S. V., Fareedullah M., Sudhakar Y., Venkateswarlu B., Kumar E. A. (2010). Current review on organophosphorus poisoning. *Archives of Applied Science Research*.

[7] Orabi S. H., Elbialy B. E., Shawky S. M. (2013). Ameliorating and hypoglycemic effects of zinc against acute hepatotoxic effect of chlorpyrifos. *Global Veterinaria*.

[8] Durak D., Uzun F. G., Kalender S., Ogutcu A., Uzunhisarcikli M., Kalender Y. (2009). Malathion-induced oxidative stress in human erythrocytes and the protective effect of vitamins C and E in vitro. *Environmental Toxicology*.

[9] Vani T., Saharan N., Roy S. D. (2012). Alteration in haematological and biochemical parameters of *Catla catla* exposed to sub-lethal concentration of cypermethrin. *Fish Physiology and Biochemistry*.

[10] Al-Damegh M. A. (2013). Toxicological impact of inhaled electric mosquito-repellent liquid on the rat: a hematological, cytokine indications, oxidative stress and tumor markers. *Inhalation Toxicology*.

[11] Petras J. M. (1981). Soman neurotoxicity. *Fundamental and Applied Toxicology*.

[12] Kadar T., Cohen G., Sahar R., Alkalai D., Shapira S. (1992). Long-term study of brain lesions following soman, in comparison to DFP and metrazol poisoning. *Human and Experimental Toxicology*.

[13] Veronesi B., Jones K., Pope C. (1990). The neurotoxicity of subchronic acetylcholinesterase (AChE) inhibition in rat hippocampus. *Toxicology and Applied Pharmacology*.

[14] Pelegrino J. R., Calore E. E., Saldiva P. H. N., Almeida V. F., Peres N. M., Vilela-de-Almeida L. (2006). Morphometric studies of specific brain regions of rats chronically intoxicated with the organophosphate methamidophos. *Ecotoxicology and Environmental Safety*.

[15] Dassanayake T., Weerasinghe V., Dangahadeniya U. (2008). Long-term event-related potential changes following organophosphorus insecticide poisoning. *Clinical Neurophysiology*.

[16] Speed H. E., Blaiss C. A., Kim A. (2012). Delayed reduction of hippocampal synaptic transmission and spines following exposure to repeated subclinical doses of organophosphorus pesticide in adult mice. *Toxicological Sciences*.

[17] Carey J. L., Dunn C., Gaspari R. J. (2013). Central respiratory failure during acute organophosphate poisoning. *Respiratory Physiology and Neurobiology*.

[18] Attia A. M., Nasr H. (2009). Dimethoate-induced changes in biochemical parameters of experimental rat serum and its neutralization by black seed (Nigella sativa L.) oil. *Slovak Journal of Animal Science*.

[19] Elhalwagy M. E. A., Darwish N. S., Zaher E. M. (2008). Prophylactic effect of green tea polyphenols against liver and kidney injury induced by fenitrothion insecticide. *Pesticide Biochemistry and Physiology*.

[20] Baars B. J., Franklin S., Ramsøy T. Z. (2013). Global workspace dynamics: cortical ‘binding and propagation’ enables conscious contents. *Frontiers in Psychology*.

[21] Ellman G. L., Courtney K. D., Andres V., Featherstone R. M. (1961). A new and rapid colorimetric determination of acetylcholinesterase activity. *Biochemical Pharmacology*.

[22] Lowe I. P., Robins E., Eyerman G. S. (1958). The fluorometric measurement of glutamic decarboxylase and its distribution in brain. *Journal of Neurochemistry*.

[23] Harrison M. J. G., Marsden C. D., Jenner P. (1979). Effect of experimental ischemia on neurotransmitter amines in the gerbil brain. *Stroke*.

[24] Zhang X., Li H., Hu S. (2006). Brain edema after intracerebral hemorrhage in rats: the role of inflammation. *Neurology India*.

[25] Kaya M., Kalayci R., Küçük M. (2003). Effect of losartan on the blood-brain barrier permeability in diabetic hypertensive rats. *Life Sciences*.

[26] Good C. A., Kramer H. M., Somogyi M. (1933). The determination of glycogen. *The Journal of Biological Chemistry*.

[27] Beutler E., Duron O., Kelly B. M. (1963). Improved method for the determination of blood glutathione. *The Journal of Laboratory and Clinical Medicine*.

[28] Sinha A. K. (1972). Colorimetric assay of catalase. *Analytical Biochemistry*.

[29] Yoshioka T., Kawada K., Shimada T., Mori M. (1979). Lipid peroxidation in maternal and cord blood and protective mechanism against activated-oxygen toxicity in the blood. *American Journal of Obstetrics & Gynecology*.

[30] Reitman S., Frankel S. (1957). A colorimetric method for the determination of serum glutamic oxalacetic and glutamic pyruvic transaminases. *American Journal of Clinical Pathology*.

[31] Belfield A., Goldberg D. M. (1971). Revised assay for serum phenyl phosphatase activity using 4-amino-antipyrine. *Enzyme*.

[32] Knedel M., Böttger R. (1967). A kinetic method for determination of the activity of pseudocholinesterase (acylcholine acyl-hydrolase 3.1. 1.8.). *Klinische Wochenschrift*.

[33] Walters M. I., Gerarde H. W. (1970). An ultramicromethod for the determination of conjugated and total bilirubin in serum or plasma. *Microchemical Journal*.

[34] Doumas B. T., Watson W. A., Biggs H. G. (1997). Albumin standards and the measurement of serum albumin with bromcresol green. *Clinica Chimica Acta*.

[35] Trinder P. (1951). A rapid method for the determination of sodium in serum. *The Analyst*.

[36] Sunderman F. W., Sunderman F. W. (1958). Studies in serum electrolytes. XXII. A rapid, reliable method for serum potassium using tetraphenylboron. *American Journal of Clinical Pathology*.

[37] Gindler E., Heth D. (1971). *Colorimetric Determination with Bound Calmagite of Magnesium in Human Blood Serum*.

[38] Richmond W. (1973). Preparation and properties of a cholesterol oxidase from Nocardia sp. and its application to the enzymatic assay of total cholesterol in serum. *Clinical Chemistry*.

[39] Burstein M., Scholnick H. R., Morfin R. (1970). Rapid method for the isolation of lipoproteins from human serum by precipitation with polyanions. *Journal of Lipid Research*.

[40] Wieland H., Seidel D. (1983). A simple specific method for precipitation of low density lipoproteins. *Journal of Lipid Research*.

[41] Fawcett J. K., Scott J. E. (1960). A rapid and precise method for the determination of urea. *Journal of Clinical Pathology*.

[42] Barham D., Trinder P. (1972). An improved colour reagent for the determination of blood glucose by the oxidase system. *Analyst*.

[43] Drabkin D. L., Austin J. H. (1932). Spectrophotometric studies I. Spectrophotometric constants for common hemoglobin derivatives in human, dog, and rabbit blood. *The Journal of Biological Chemistry*.

[44] Trinder P. (1969). Determination of blood glucose using an oxidase-peroxidase system with a non-carcinogenic chromogen. *Journal of Clinical Pathology*.

[45] Hull R., Hirsh J., Jay R. (1982). Different intensities of oral anticoagulant therapy in the treatment of proximal-vein thrombosis. *The New England Journal of Medicine*.

[46] Henry R., Cannon D., Winkelman J. (1974). *Clinical Chemistry: Principles and Technics*.

[47] Konitzer K., Voigt S. (1963). Direct determination of ammonium in blood and tissue extracts by means of the phenol by chlorite reaction. *Clinica Chimica Acta; International Journal of Clinical Chemistry*.

[48] Patil J. A., Patil A. J., Govindwar S. P. (2003). Biochemical effects of various pesticides on sprayers of grape gardens. *Indian Journal of Clinical Biochemistry*.

[49] Kavitha B., Shruthi S., Rai S. P., Ramachandra Y. (2011). Phytochemical analysis and hepatoprotective properties of *Tinospora cordifolia* against carbon tetrachloride-induced hepatic damage in rats. *Journal of Basic and Clinical Pharmacy*.

[50] Jain N. K., Singhai A. K. (2011). Protective effects of *Phyllanthus acidus* (L.) Skeels leaf extracts on acetaminophen and thioacetamide induced hepatic injuries in Wistar rats. *Asian Pacific Journal of Tropical Medicine*.

[51] Oh S. W., Lee E. S., Kim S. (2013). Bilirubin attenuates the renal tubular injury by inhibition of oxidative stress and apoptosis. *BMC Nephrology*.

[52] Bortolussi G., Codarin E., Antoniali G. (2015). Impairment of enzymatic antioxidant defenses is associated with bilirubin-induced neuronal cell death in the cerebellum of Ugt1 KO mice. *Cell Death & Disease*.

[53] Mossa A.-T. H., Refaie A. A., Ramadan A. (2011). Effect of exposure to mixture of four organophosphate insecticides at no observed adverse effect level dose on rat liver: the protective role of vitamin C. *Research Journal of Environmental Toxicology*.

[54] Heneghan C., Ward A., Perera R. (2012). Self-monitoring of oral anticoagulation: systematic review and meta-analysis of individual patient data. *The Lancet*.

[55] Amin K. A., Hashem K. S. (2012). Deltamethrin-induced oxidative stress and biochemical changes in tissues and blood of catfish (Clarias gariepinus): antioxidant defense and role of alpha-tocopherol. *BMC Veterinary Research*.

[56] Anusha M., Venkateswarlu M., Prabhakaran V., Taj S. S., Kumari B. P., Ranganayakulu D. (2011). Hepatoprotective activity of aqueous extract of *Portulaca oleracea* in combination with lycopene in rats. *Indian Journal of Pharmacology*.

[57] Acker C. I., Nogueira C. W. (2012). Chlorpyrifos acute exposure induces hyperglycemia and hyperlipidemia in rats. *Chemosphere*.

[58] Nagaraju R., Joshi A. K. R., Rajini P. S. (2015). Organophosphorus insecticide, monocrotophos, possesses the propensity to induce insulin resistance in rats on chronic exposure. *Journal of Diabetes*.

[59] Southam A. D., Lange A., Hines A. (2011). Metabolomics reveals target and off-target toxicities of a model organophosphate pesticide to roach (*Rutilus rutilus*): implications for biomonitoring. *Environmental Science & Technology*.

[60] Pakzad M., Fouladdel S., Nili-Ahmadabadi A. (2013). Sublethal exposures of diazinon alters glucose homostasis in Wistar rats: biochemical and molecular evidences of oxidative stress in adipose tissues. *Pesticide Biochemistry and Physiology*.

[61] Malekirad A. A., Faghih M., Mirabdollahi M., Kiani M., Fathi A., Abdollahi M. (2013). Neurocognitive, mental health, and glucose disorders in farmers exposed to organophosphorus pesticides. *Archives of Industrial Hygiene and Toxicology*.

[62] Ismail S. M. (2013). Protective effects of vitamin C against biochemical toxicity induced by malathion pesticides in male albino rat. *Journal of Evolutionary Biology Research*.

[63] Mehra B., Sharma P., Kaushik U., Joshi S. (2014). Effect of fytolan on haematology and serum parameters of male albino rats. *World Journal of Pharmaceutical Sciences*.

[64] Hundekari I. A., Suryakar A. N., Rathi D. B. (2013). Acute organo-phosphorus pesticide poisoning in North Karnataka, India: oxidative damage, haemoglobin level and total leukocyte. *African Health Sciences*.

[65] Muthulingam M., Mohandoss P., Indra N., Sethupathy S. (2010). Antihepatotoxic efficacy of Indigofera tinctoria (Linn.) on paracetamol induced liver damage in rats. *International Journal of Pharmaceutical and Biomedical Research*.

[66] Hassan H. A., Yousef M. I. (2010). Ameliorating effect of chicory (*Cichorium intybus* L.)-supplemented diet against nitrosamine precursors-induced liver injury and oxidative stress in male rats. *Food and Chemical Toxicology*.

[67] Mansour S. A., Mossa A.-T. H. (2010). Oxidative damage, biochemical and histopathological alterations in rats exposed to chlorpyrifos and the antioxidant role of zinc. *Pesticide Biochemistry and Physiology*.

[68] Elzoghby R. R., Hamoda A. F., Abed-Ftah A., Farouk M. (2014). Protective role of vitamin C and green tea extract on malathion-induced hepatotoxicity and nephrotoxicity in rats. *American Journal of Pharmacology and Toxicology*.

[69] Sundaram V., Manne V., Al-Osaimi A. M. S. (2014). Ascites and spontaneous bacterial peritonitis: recommendations from two United States centers. *Saudi Journal of Gastroenterology*.

[70] Pohl H. R., Wheeler J. S., Murray H. E. (2013). Sodium and potassium in health and disease. *Interrelations between Essential Metal Ions and Human Diseases*.

[71] Gumz M. L., Rabinowitz L., Wingo C. S. (2015). An integrated view of potassium homeostasis. *The New England Journal of Medicine*.

[72] Mossalam H. H., Abd-El Aty O. A., Morgan E. N., Youssaf S., Mackawy A. M. H. (2011). Biochemical and ultra structure studies of the antioxidant effect of aqueous extract of hibiscus sabdariffa on the nephrotoxicity induced by organophosphorous pesticide (malathion) on the adult albino rats. *Journal of American Science*.

[73] Weiner I. D., Mitch W. E., Sands J. M. (2015). Urea and ammonia metabolism and the control of renal nitrogen excretion. *Clinical Journal of the American Society of Nephrology*.

[74] Giuliani C., Peri A. (2014). Effects of Hyponatremia on the Brain. *Journal of Clinical Medicine*.

[75] Rivera-Espinosa L., Floriano-Sánchez E., Pedraza-Chaverrí J. (2013). Contributions of microdialysis to new alternative therapeutics for hepatic encephalopathy. *International Journal of Molecular Sciences*.

[76] Ahmed M. A. E., Ahmed H. I., El-Morsy E. M. (2013). Melatonin protects against diazinon-induced neurobehavioral changes in rats. *Neurochemical Research*.

[77] Jones D. C., Miller G. W. (2008). The effects of environmental neurotoxicants on the dopaminergic system: a possible role in drug addiction. *Biochemical Pharmacology*.

[78] Zhang J., Dai H., Deng Y. (2015). Neonatal chlorpyrifos exposure induces loss of dopaminergic neurons in young adult rats. *Toxicology*.

[79] Cichoż-Lach H., Michalak A. (2013). Current pathogenetic aspects of hepatic encephalopathy and noncirrhotic hyperammonemic encephalopathy. *World Journal of Gastroenterology*.

[80] Moghaddam B., Javitt D. (2012). From revolution to evolution: the glutamate hypothesis of schizophrenia and its implication for treatment. *Neuropsychopharmacology*.

[81] Sidoryk-Wegrzynowicz M., Aschner M. (2013). Manganese toxicity in the central nervous system: the glutamine/glutamate-*γ*-aminobutyric acid cycle. *Journal of Internal Medicine*.

[82] Avraham Y., Grigoriadis N. C., Poutahidis T. (2011). Cannabidiol improves brain and liver function in a fulminant hepatic failure-induced model of hepatic encephalopathy in mice. *British Journal of Pharmacology*.

[83] Häussinger D., Görg B. (2010). Interaction of oxidative stress, astrocyte swelling and cerebral ammonia toxicity. *Current Opinion in Clinical Nutrition and Metabolic Care*.

[84] Noaishi M. A., Afify M. M., Abd Allah A. A. (2013). Study the inhalation exposure effect of pesticides mixture in the white rat. *Nature & Science*.

[85] Falcão H., Japiassú A. M. (2011). Albumin in critically ill patients: controversies and recommendations. *Revista Brasileira de Terapia Intensiva*.

[86] Cataudella E., Malaguarnera G., Gagliano C. (2012). Pesticides exposure and the management of acute hepatic injury. *Acta Medica Mediterranea*.

[87] Collombet J.-M. (2011). Nerve agent intoxication: recent neuropathophysiological findings and subsequent impact on medical management prospects. *Toxicology and Applied Pharmacology*.

[88] Osman A., Mastan S. A., Rabia Banu S., Indira P. (2015). Sub-lethal effect of cypermethrin on acetylcholinesterase (AChE) activity and acetylcholine (Ach) content in selected tissues of Channa striatus (Bloch.). *Journal of Toxicology and Environmental Health Sciences*.

[89] Pohanka M. (2014). Inhibitors of acetylcholinesterase and butyrylcholinesterase meet immunity. *International Journal of Molecular Sciences*.

[90] Elhalwagy M. E. A., Darwish N. S., Shokry D. A. (2015). Garlic and *α* lipoic supplementation enhance the immune system of albino rats and alleviate implications of pesticides mixtures. *International Journal of Clinical and Experimental Medicine*.

[91] Franco J. L., Posser T., Mattos J. J. (2009). Zinc reverses malathion-induced impairment in antioxidant defenses. *Toxicology Letters*.

[92] Larsen F. S., Gottstein J., Blei A. T. (2001). Cerebral hyperemia and nitric oxide synthase in rats with ammonia-induced brain edema. *Journal of Hepatology*.

[93] Scott T. R., Kronsten V. T., Hughes R. D., Shawcross D. L. (2013). Pathophysiology of cerebral oedema in acute liver failure. *World Journal of Gastroenterology*.

[94] Penchalamma R., Jacob D. P. (2014). Dimethoate on protein metabolic profiles in rat kidney. *Weekly Science Research Journal*.

[95] Bosoi C. R., Yang X., Huynh J. (2012). Systemic oxidative stress is implicated in the pathogenesis of brain edema in rats with chronic liver failure. *Free Radical Biology and Medicine*.

[96] Lemberg A., Fernandez M. A. (2009). Hepatic encephalopathy, ammonia, glutamate, glutamine and oxidative stress. *Annals of Hepatology*.

[97] Bajaj J. S. (2014). The role of microbiota in hepatic encephalopathy. *Gut Microbes*.

[98] Rahman T., Hosen I., Islam M. M. T., Shekhar H. U. (2012). Oxidative stress and human health. *Advances in Bioscience and Biotechnology*.

[99] Badade Z., Rastogi S., Singh S. (2013). Antioxidant status and oxidative stress in organophosphate pesticide poisoning. *Journal of Dental and Medical Sciences*.

[100] Diab A. E.-A. A., El-Aziz E.-S. A. A., Hendawy A. A., Zahra M. H., Hamza R. Z. (2012). Antioxidant role of both propolis and ginseng against neurotoxicity of chlorpyrifos and profenofos in male rats. *Life Science Journal-Acta Zhengzhou University Overseas*.

[101] Ahmed M. M., Zaki N. I. (2009). Assessment the ameliorative effect of pomegranate and rutin on chlorpyrifos-ethyl-induced oxidative stress in rats. *Nature and Science*.

[102] Al-Othman A. M., Al-Numair K. S., El-Desoky G. E. (2011). Protection of *α*-tocopherol and selenium against acute effects of malathion on liver and kidney of rats. *African Journal of Pharmacy and Pharmacology*.

[103] Elhalwagy M. E. A., Zaki N. I. (2009). Comparative study on pesticide mixture of organophosphorus and pyrethroid in commercial formulation. *Environmental Toxicology and Pharmacology*.

[104] Heikal T. M., El-Sherbiny M., Hassan S. A., Arafa A., Ghanem H. Z. (2012). Antioxidant effect of selenium on hepatotoxicity induced by chlorpyrifos in male rats. *International Journal of Pharmacy and Pharmaceutical Sciences*.

[105] Baconi D. L., Bârcă M., Manda G., Ciobanu A.-M., Bălălău C. (2013). Investigation of the toxicity of some organophosphorus pesticides in a repeated dose study in rats. *Romanian Journal of Morphology and Embryology*.

[106] Newairy A., Abdou H. (2013). Effect of propolis consumption on hepatotoxicity and brain damage in male rats exposed to chlorpyrifos. *African Journal of Biotechnology*.

[107] Owoeye O., Edem F. V., Akinyoola B. S., Rahaman S., Akang E. E., Arinola G. O. (2012). Histological changes in liver and lungs of rats exposed to dichlorvos before and after vitamin supplementation. *European Journal of Anatomy*.

